# A Reflection on Community Research and Action as an Evolving Practice

**DOI:** 10.1007/s42822-021-00083-x

**Published:** 2021-11-12

**Authors:** Stephen B. Fawcett

**Affiliations:** Center for Community Health and Development, Life Span Institute, University of Kansas, 4082 Dole Center, 1000 Sunnyside Ave, Lawrence, KS 66045 USA

**Keywords:** Community research, Participatory research, Capacity building, Behavioral science, Community psychology, Public health

## Abstract

Community research and action is an evolving field of practice with multiple influences. Its varied ways of knowing and doing reflect recombined elements from different disciplines, including behavioral science, community psychology, public health, and community development. This article offers a personal reflection based on my evolving practice over nearly 50 years. The focus is on three types of influence: (a) engaging with different communities, fields, and networks (e.g., discovering shared values, diverse methods); (b) building methods and capabilities for the work (e.g., methods for participatory research, tools for capacity building); and (c) partnering for collaborative research and action, locally and globally. This story highlights the nature of the field’s evolution as an increasing variation in methods. Our evolving practice of community research and action—individually and collectively—emerges from the recombination of ideas and methods discovered through engagement in a wide variety of contexts.

Gradually, the observer realizes that these organisms are connected with each other, not linearly, but in a net-like, entangled fabric.

—Alexander von Humboldt, German naturalist and explorer

In our professional lives, we follow branches from a field of origin—perhaps behavioral science or public health—into other related fields. In exchanges with others with different training and experience, we share ideas and methods that alter our practice and enrich our collective work. Like the “entangled life” of fungi (Sheldrake, 2020), we are connected in a web of relationships through which ideas and methods are shared and recombined in novel forms.

Community research and action is an evolving practice with multiple influences. Its varied activities result from exposure to, and selection for, different ways of knowing and doing. Recombined elements of research and practice reflect influences from different disciplines, including applied behavioral science, community psychology, public health, applied anthropology, urban planning, and community development, among others. For instance, if we are trained in behavioral science, we may especially value systematic methods of measurement and intervention. Exposure to community-oriented disciplines, such as community psychology or community development, may add an emphasis on participatory approaches, as represented in community-based participatory research and community engagement in designing and implementing strategies for action. Subsequent exposure to public health methods may add systems approaches and methods for changing conditions that affect health and health equity.

This article offers a reflection on the evolving practice of community research and action. Illustrated with my 50-plus years of experience, it focuses on three important mechanisms: (a) engaging with different communities, fields, and networks; (b) building methods and capabilities for the work; and (c) partnering for collaborative research and action.

## Background and Context for Learning and Contributing

### Personal Background

Personal backgrounds shape our openness to engaging people and seeing issues and concerns, as well as the possible ways of addressing them. My family and cultural background as an Irish Catholic led to exposure to Catholic social teaching. This called for a preferential option for the poor, solidarity with those who are marginalized, and a duty to pursue justice and address inequities. My undergraduate training in biology led to a lifelong interest in understanding mechanisms—how things work—including how community processes can produce changes in community conditions and outcomes.

After college, I joined the Volunteers in Service to America, where I lived and worked in low-income public housing in Kansas City. Going door to door, I met with and listened to people talk about what mattered to them. Through the kindness and wisdom of local guides (especially community leaders Myrtle Carter, Leotha Pinckney, and Freddie Coleman), I was led to see the community’s strengths and weaknesses, threats to progress, and opportunities for improvement through collective action. Together, we organized a tenants’ association to address community-determined concerns related to housing, education, violence, and building a good community for raising children. This experience in community organizing led to an appreciation for understanding the felt concerns of people in communities and their reality-based ideas for taking action.

During subsequent graduate/PhD training in applied behavioral science, I studied methods for measuring behavior and creating interventions and environmental conditions that can promote socially important behaviors and outcomes. I learned about methods to analyze personal and environmental factors contributing to problems and goals, and to design and implement effective interventions. Guides and mentors (e.g., Mont Wolf, Todd Risley, Jim Sherman, Keith Miller, and Dick Schiefelbusch) helped me see how the field could further systematic work in community research and action.

Each of us has our own combination of background, training, and experience that prepare us for the work of community research and action. However, curiosity and a desire for impact may lead us to search for additional methods that complement those acquired in early training and experience.

### Context and Base for Learning and Contributing

Each of us has a different context for learning and contributing to community well-being. For many of us, this involves work at the individual level, listening and caring for family members, neighbors, and coworkers. Others may have public service roles or professional responsibilities related to improving conditions at the level of organizations, whole communities, or broader systems.

My professional mission has been to help understand and improve how people and organizations can work together to change conditions for improved health, well-being, and equity (Fawcett, Schultz, et al., 2010b). In my role as a professor in a research university, I had the privilege of working in the field of community research and action. In my teaching, I tried to guide students in their learning about applied behavioral research and building healthy communities. With colleagues, I established an undergraduate program in community health and development and a joint PhD/MPH program (PhD in applied behavioral science, master’s in public health).

My primary base for learning and contributing was as founding director (in 1975) of the Work Group/Center for Community Health and Development at the University of Kansas (KU; https://communityhealth.ku.edu/). With generations of graduate students and colleagues, we sought to achieve the center’s mission of promoting community health and development through collaborative research, teaching, and public service. Since 2004, our KU center has valued its designation as a World Health Organization (WHO) Collaborating Centre for Community Health and Development, thereby connecting us with global partners with whom to exchange, learn, and contribute.

## Mechanisms for Evolving Practice: Engagement, Methods Building, and Partnerships

Community work fosters humility. This is true because we so often fall short of the desired goal of achieving improved conditions and outcomes. This may lead us to search for people and methods to achieve a better result and to have a broader impact. In this section, I consider three such mechanisms for evolving practice: (a) engaging with different communities, fields, and networks; (b) building methods and capabilities; and (c) partnering for collaborative research and action.

### Engagement With Different Communities, Fields, and Networks

Through involvement in varied contexts, we are exposed to different people and ideas, values, and methods. In my own work, I have had the opportunity to learn from and with communities locally, nationally, and globally. We see countless examples of people working together to improve conditions and outcomes. For instance, we can learn from those working in community organizations throughout the United States (Fawcett, 1999) or from community health workers engaged in different parts of the world (Fawcett, Abeykoon, et al., 2010a). Working in solidarity with these colleagues, we note shared values in community work—for engagement, empowerment, equity, and attention to broader determinants of health and well-being.

Engagement with different disciplines and fields brings exposure to diverse methods for community research and action. If we bring only a critical eye from narrow training in a single discipline, we may fail to see the potential contribution of new methods to help understand the situation and improve conditions. By contrast, if we bring an appreciative stance, we can see how methods found in other contexts and disciplines can expand our approaches for engagement, assessment, planning, intervention, and evaluation of efforts.

My own experience reflects a layering of disciplinary influences over time. From 1969 to 1971, work in community organizing brought an appreciation for starting with the felt needs of local people and other valuable approaches in community development. Beginning in 1975, my teaching and research were grounded in PhD training in applied behavioral science. Particular strengths of this field include methods to measure behavior and assess conditions, analyze personal and environmental factors contributing to problems and goals, and design and implement effective interventions.

In pursuit of additional methods to inform community work, I sought out potential guides in the field of community psychology. Beginning in the late 1970s, this has been a career-long engagement, with attempts to integrate work in behavioral community psychology (e.g., Fawcett et al., 1980). Through the generosity of guides in community psychology (e.g., Lenny Jason, Rick Price, Tom Wolff, and Bill Berkowitz), I discovered inspiring people and work and new methods for community research and action. By seeking an integration of the fields of applied behavioral science and community psychology—a form of behavioral community psychology—we tried to bridge important values and standards of these two disciplines (Fawcett, 1990, 1991).

Beginning in the early 1990s, our work with the Kansas Health Foundation and a subsequent endowed professorship reoriented our center’s work to the field of public health. Through guides in public health (e.g., Marni Vliet, Larry Green, Marshall Kreuter, Michael McGinnis, and Bobbie Berkowitz), we discovered the shared values of social justice, evidence-based practice, and commitment to creating conditions for health and equity that are the pillars of this discipline.

Beginning in 2004 and still ongoing, our center was designated by the WHO as a Collaborating Centre for Community Health and Development. This allowed us to learn and contribute with colleagues from around the world, with encouragement and support from guides in global health (e.g., Bill Foege, Gauden Galea, Alfonso Contreras, Gerry Eijkemans, Peter Phori, and Rima Afifi). The WHO Collaborating Centre’s two primary objectives—building capacity for the work of community health and development and expanding the evidence base for collaborative action—continue to be a focus for our broader KU center.

These and other disciplines, and related interest groups and networks, have created a rich web of opportunities for many of us to learn how to engage in community research and action.

### Building Methods and Capabilities for the Work

Every practitioner seeks to discover and adapt methods to make the work of promoting community health and development more effective. We develop tools and protocols, such as for assessment or intervention, to make the work easier for ourselves and others. We build capabilities, such as for workforce development or participatory evaluation, to enable others to do this work—without us, in their different contexts, long after we are gone.

Our KU center has focused its development efforts on two strategic capabilities: (a) tools for capacity building and (b) methods for participatory research and monitoring and evaluation.

#### Tools for Capacity Building: The Community Tool Box and Action Toolkits

In 1995, a team of colleagues (myself, Jerry Schultz, Vincent Francisco, Bill Berkowitz, and Tom Wolff) began building the Community Tool Box (http://ctb.ku.edu/). That work continues with our KU center, under the leadership of Christina Holt. The Community Tool Box is now a massive (over 7,000-page), free, and open-source collection of tools for building capacity for this work. It features hundreds of learning modules—including task analyses, rationales, and application examples—for skills related to promoting community health and development. Learning modules aim to build capacity for core competencies in community research and action, including engagement, assessment, planning, intervention, advocacy, and evaluation. Available in English and Spanish, and partially in Arabic and Farsi, the open-source Community Tool Box reached over 6,000,000 unique users last year.

In recent years, we have also developed customized capacity-building resources, known as Action Toolkits, with a number of different partners. These online resources mix other content sources with curated content from the Community Tool Box—including its task analyses—to build skills for implementing a partner’s framework for action. For instance, the African Health Action Toolkit (https://who-afro.ctb.ku.edu), developed with partners at the WHO Regional Office for Africa, is intended to build capacity for addressing social determinants of health and furthering sustainable development goals in the region. The Healthy Cities Action Toolkit (https://paho.ctb.ku.edu/), developed with the WHO/Pan American Health Organization (PAHO) Regional Office for the Americas and available in English and Spanish, aims to support efforts to promote healthy cities in the Americas. Partnering with a state health department, we built the Kansas Healthy Communities Action Toolkit (https://ksactiontoolkit.ctb.ku.edu/) to further health-equity work. Other partnerships have produced an array of Action Toolkits, including those for improving community health, promoting racial justice, strengthening democratic action, and promoting compassionate communities.

#### Methods for Participatory Research and Monitoring and Evaluation

Our center also invested in developing a capability for monitoring and evaluation (M&E) that would allow us to work with partners locally and globally. The technology for this M&E system, known as the Community Check Box Evaluation System, supports the documentation of the intervention and participatory sensemaking to reflect on patterns in the data (Fawcett et al., 2017). With partners, we have used this M&E system to help understand and improve a variety of collaborative efforts.

Participatory M&E—a form of participatory action research—holds promise for understanding and addressing a variety of health and development issues. Capabilities that make this easier can be helpful in facilitating partnerships for community research. For instance, we have used this methodology to support evaluations of initiatives to (a) promote community health and development (e.g., Fawcett et al., 2016), (b) enhance care coordination for those with low incomes (e.g., Hassaballa et al., 2015), (c) prevent the spread of Ebola in Liberia (e.g., Munodawafa et al., 2018), (d) provide a health-systems response to COVID-19 (Holt et al., 2021), and (e) respond to COVID-19 in the WHO Africa Region. For instance, in the latter example, the WHO Regional Office for Africa used the M&E system to document the unfolding of COVID-19 response activities in African countries, support country partners’ reflections on patterns, and adjust its technical support for country efforts (Phori et al., manuscript under revision).

The sensemaking protocol of the M&E system enables stakeholders—including those most affected and those responsible—to construct their own meaning of the data. They do so by systematically reflecting on (a) what we are seeing (i.e., in data patterns), (b) what it means (e.g., identifying factors and critical events associated with increases/decreases in measures), and (c) what the implications for adjustment and improvement are. We have seen the value of protocols for participatory M&E, and the related use of the Community Check Box Evaluation System, in an array of partnerships.

By building tools and platforms for making the work easier and more effective—and more participatory—we can strengthen engagement with partners and extend the learning, reach, and impact of our efforts.

### Partnering for Collaborative Research and Action

Collaborative partnerships involve a sharing of resources, responsibilities, risks, and rewards (Himmelman, 2002). This requires trust and the experience borne of respectful engagement with different communities and fields. Capabilities that make the work easier and more effective, such as those for capacity building or evaluation, make it more likely that partners will choose to work together.

Our center has had the privilege of working with an array of partners on a variety of initiatives, typically in the roles of training, technical support, and evaluation. For instance, locally, in an over decade-long partnership with the Latino Health for All Coalition, we have provided technical support and evaluation for the coalition’s efforts to promote physical activity, healthy nutrition, and access to health services (e.g., Collie-Akers et al., 2013), including enhancing health access and culturally competent health services (Fawcett et al., 2018) and enrolling underserved groups in affordable health insurance (e.g., Fawcett, Sepers, et al., 2015b). In a partnership with a state health department, we designed and supported the implementation of a maternal and child health M&E system to document and improve system changes related to improving conditions for population-level maternal and child health.

Nationally, we have used this systematic M&E capability to document and characterize the intensity of community efforts to prevent childhood obesity in the national Healthy Communities Study that involved over 300 communities (Fawcett, Collie-Akers, et al., 2015a; Frongillo et al., 2017; Strauss et al., 2018). As evaluators of the Bristol Meyers Squibb Foundation’s national Together on Diabetes Program, we also used this M&E system to support the accountability and quality improvement of multiple partners working to address equity issues in diabetes care (e.g., Hassaballa et al., 2015).

Globally, in partnership with the WHO Regional Office for Africa, we have worked to expand the evidence base for how communities and countries respond to communicable disease outbreaks such as Ebola (e.g., Munodawafa et al., 2018). In a current project, the Community Check Box serves as the infrastructure for a WHO AFRO effort to document and better understand country-level responses to COVID-19 within the Africa region. This project uses the participatory sensemaking protocol to identify factors that enabled and impeded the response and associated effects on new cases of COVID-19 (Phori et al., manuscript under revision).

Locally, and globally, the Community Tool Box—with over 6,000,000 unique users—has the broadest reach of the center’s projects and capabilities (Holt et al., 2013). It builds capacities to provide training and technical support for the workforce, including assessment, planning, intervention, advocacy, and evaluation. Its free and open-source materials support the work of millions of learners and practitioners from over 200 countries—including those working in their own communities and organizations, and in government, nongovernmental organizations, and civil society. Action Toolkits, based on the Community Tool Box, help serve the customized capacity-building needs of partners with extensive reach, such as the WHO’s Regional Office for the Americas/PAHO (https://paho.ctb.ku.edu/), Regional Office for Africa (https://who-afro.ctb.ku.edu), and Regional Office for the Western Pacific (https://who-wpro.ctb.ku.edu/engage/).

## Conclusion: Our Shared Story of Exchange and Variation

This article posits three mechanisms by which community research and practice evolves: (a) engaging with different communities, fields, and networks; (b) building methods and capabilities for the work; and (c) partnering for collaborative research and action. This personal reflection tells a story of evolution—of change and adaptation, of selection and recombination of elements, and ultimately of variation. This process of evolution seems to hold for us individually, and collectively as a community of practitioners developing and adapting ways of doing the work.

As with biological evolution, chance and opportunity play an important part in variation. For instance, although we may seek guides to help show the way in different communities and fields, they may not be available to us. Although we might hope to build capabilities to make the work easier, we may not find the resources to do so. Despite our interest in partnerships, our modest relationships and limited experience may not enable us to forge them. In addition, as with biological mutations, not all variations in community methods are good; there is a risk that change may not equal improvement.

Paleontologist and evolutionary scientist Stephen Jay Gould (1996) noted in *Full House* that the story line for biological evolution is more variation than progress. Chance events and related differential exposures, vulnerabilities, and capabilities lead to life-forms of great variety. Evolutionary history shows more evidence of variation than improved functioning. This might also apply to the field of community research and action. Analogously, rather than look for one approach as the pinnacle, we might do better to appreciate the accumulating variation that emerges from our collective engagements, methods building, and partnerships.

This personal reflection highlights the mechanisms that increase variation—and perhaps some progress—in the field of community research and action. Our evolving practice emerges from exchange among partners and the recombination of ideas and methods discovered through engagement with different communities and fields. This is the work of seekers—those with curiosity and openness to new methods and adaptations that may have a relative advantage. May we have “entangled” lives, ones that are enriched by a web of relationships through which we learn, change, and improve our collective contributions to community health, development, and equity.
